# Supplementary material for: Food Reward after a Traditional Inuit or a Westernised Diet in an Inuit Population in Greenland
Source: Nutrients. 2022 Jan 27;14(3):561. doi: 10.3390/nu14030561 (PMC8839061; doi:10.3390/nu14030561)
Supplement: Supplementary file 1 [file nutrients-14-00561-s001.zip › nutrients-1516898-supplementary.pdf]

Table S1. Food reward after four weeks on a traditional or western diet as well as overall results for food reward among all participants.

	**Traditional**	**Westernized**	***Difference (95% CI)***	***P-value***	**Overall**
n	20	24			44
	**Explicit liking (*n*=44)**				
Sweet bias	-10.6 (-15.8, -1.1)	-11.6 (-21.6, -4.8)	*-4.0 (-13.0; 5.0*)	*0.39*	-11.5 (-19.4, -3.4)
Fat bias	-15.9 (-21.9, -3.7)	-24.4 (-30.4, -2.2)	*-4.8 (-15.2; 5.7]*	*0.37*	-17.4 (-29.3, -2.5)
HFSA	57.8 (44.4, 68.2)	50.2 (42.2, 75.8)	*3.8 (-8.8; 16.5*)	*0.56*	54.2 (42.6, 71.1)
HFSW	19.5 (12.8, 28.2)	26.2 (9.7, 35.8)	*-3.4 (-15.3; 8.4*)	*0.57*	22.1 (11.4, 35.6)
LFSA	53.5 (38.2, 63.0)	54.6 (34.4, 63.2)	*5.3 (-7.9; 18.4*)	*0.43*	53.5 (34.4, 63.2)
LFSW	63.0 (39.8, 79.2)	61.4 (51.6, 75.8)	*4.6 (-8.2; 17.4*)	*0.48*	61.4 (45.8, 77.1)
	**Implicit wanting (*n=43*)**				
Sweet bias	-18.4 (-45.2, 1.4)	-21.8 (-40.0, -10.6)	*-12.1 (-28.7; 4.5*)	*0.15*	-21.5 (-44.2, -3.5)
Fat bias	-19.7 (-30.4, -6.6)	-31.3 (-50.0, -17.0)	*-8.7 (-25.5; 8.2*)	*0.31*	-25.9 (-43.7, -14.0)
HFSA	19.8 (7.4, 27.2)	21.6 (11.2, 32.7)	*14.1 (-1.1; 29.2*)	*0.068*	21.6 (9.4, 32.5)
HFSW	-38.7 (-52.7, -32.6)	-52.4 (-58.6, -43.1)	*-22.7 (-43.1; -2.4*)	*0.029*	-48.1 (-56.9, -34.4)
LFSA	8.0 (-12.3, 26.8)	3.0 (-5.2, 12.1)	*-2.0 (-15.1; 11.2*)	*0.77*	3.8 (-11.2, 17.6)
LFSW	21.1 (-10.7, 31.1)	23.6 (14.1, 37.6)	*10.6 (-2.7; 24*)	*0.11*	22.8 (9.0, 36.8)

Food reward is presented as median [IQR]. The statistical differences between diet groups was examined using a multiple linear regression model. The models were adjusted for sex and age. Measures of food reward after four weeks following either a Traditional Inuit diet or a Westernized diet as well as the differences between these diets were first assessed. Explicit liking and implicit wanting for foods did not differ for participants following either of the two diets. However, participants' implicit wanting for high-fat sweet foods was lower for the group following a Traditional Inuit diet compared to participants following a Westernized diet. HFSA, high-fat savory foods; LFSA, low-fat savory foods; HFSW, high-fat sweet foods; LFSW, low-fat sweet foods
